# Immunomodulators and Immunosuppressants for Japanese Patients with Ulcerative Colitis

**DOI:** 10.5402/2011/194324

**Published:** 2011-05-25

**Authors:** Shigeki Bamba, Tomoyuki Tsujikawa, Masaya Sasaki, Yoshihide Fujiyama, Akira Andoh

**Affiliations:** ^1^Division of Gastroenterology, Shiga University of Medical Science, Shiga 521-2192, Japan; ^2^Division of Clinical Nutrition, Shiga University of Medical Science, Shiga 521-2192, Japan; ^3^Division of Mucosal Immunology, Graduate School of Medicine, Shiga University of Medical Science, Shiga 521-2192, Japan

## Abstract

Ulcerative colitis (UC) is characterized by a long-standing chronic course with remissions and exacerbations. Previously, patients do not respond to 5-aminosalicylic acid compounds and corticosteroids are considered for colectomies, however, in recent years, alternative treatments emerged for steroid-refractory or steroid-dependent UC. In this review article, we focus on immunomodulators (such as azathioprine [AZA] and 6-mercaptopurine [6-MP]) and immunosuppressants (such as cyclosporine A [CSA] and tacrolimus [FK506]) for steroid-refractory or steroid-dependent ulcerative colitis. The characteristics, efficacy and adverse effects of these drugs are outlined. Although the randomized trial of FK506 is conducted in Japan, the clinical data of CSA in Japanese patients are limited. The short-, mid- and long-term follow-ups of CSA administration in Japanese patients are discussed. As for thipurine drugs, the clinical importance of multidrug-resistance protein 4 (MRP4) in Japanese patients is highlighted.

## 1. Introduction

Ulcerative colitis (UC) is characterized by a long-standing chronic course with remissions and exacerbations. Approximately 15% of patients have severe attacks requiring hospitalization at some time during their disease course. These patients are traditionally treated with intravenous corticosteroids, with a response rate of approximately 60%. Previously, patients do not respond to 5-aminosalicylic acid compounds and corticosteroids are considered for colectomies, however, in recent years, alternative treatments emerged for steroid-refractory or steroid-dependent UC; immunomodulators (such as azathioprine [AZA] and 6-mercaptopurine [6-MP]), immunosuppressants (such as cyclosporine A [CSA] and tacrolimus [FK506]), and anti-TNF*α*-antibody (infliximab). 

In this part, we focus on immunomodulators and immunosuppressants. Unfortunately we have never used methotrexate before and have little experience on anti- TNF*α*-antibody. Therefore these two agents are excluded. Immunomodulators and immunosuppressants are essential for the induction and maintenance of UC. For induction therapy, CSA and FK506 are mainly used because of their rapid onset of effectiveness. On the other hand, AZA and 6-MP (sometimes FK506) are used in the maintenance therapy of UC. AZA and 6-MP have a slow onset of action and are therefore usually ineffective in acute disease flare-ups. In this paper, the characteristics and usage of these drugs are described focusing on Japanese patients.

## 2. Calcineurin Inhibitors (CNI)

### 2.1. Cyclosporin A (CSA)

CSA blocks the calcium-dependent signal-transduction pathway emanating from the T-cell receptor, thereby inhibiting the action of helper T cells. CSA is administrated for the patients with graft-versus-host disease after allogeneic bone marrow transplantation or the patients with renal transplant. As for ulcerative colitis, the induction of CSA for severe, steroid-refractory UC has provided an effective medical alternative to patients previously faced with only surgical options. Uncontrolled trials [1, 2] and controlled trials [3] established the efficacy of short-term CSA use as “rescue therapy” in severe UC. Lichtiger et al. reported intravenous CSA followed by oral therapy showed an initial response rate of 82% within a mean of 7 days versus 0% in a group that received steroids alone. Quality of life analyses comparing UC patients treated with CSA to those who underwent colectomies have shown that CSA patients consistently score as well as, or better than, their surgical counterparts [4]. We have started CSA administration for refractory UC patients since 1999 according to Lichtiger's method [3].

Prior to CSA administration, cytomegalovirus infection should be checked by using cytomegalovirus antigenemia (C7-HRP), PCR, or immunohistochemistry for cytomegalovirus from endoscopic biopsy specimens. If cytomegalovirus infection is suspected, dose reduction of prednisolone (PSL) and administration of ganciclovir are recommended. In our hospital, CSA was administrated by continuous infusions with starting doses of 2 mg/kg per day for a maximum of 14 days. Serum CSA levels were monitored three times a week during infusion therapy and the infusion dose was altered by aiming for 350–450 ng/mL. After successful continuous CSA infusions, we switched from continuous infusions to oral dosing. Total oral daily doses were double those of continuous daily infusions. Trough serum levels were monitored and the dose of CSA was adjusted to trough serum levels of 100–200 ng/mL. 

We have reviewed medical charts and the recent followup of 41 patients who had been administrated CSA for disease flare-ups between 1999 and 2009. The response rate at 2 weeks after CSA administration was 71%. CSA responders were defined as those with a 50-point decrease during continuous CSA infusions in Seo's complex integrated disease activity index (CIDAI) [5]. A background analysis was performed on 37 patients without early CSA discontinuation and revealed three prognostic factors: (i) more than 10,000 mg of PSL used before starting CSA; (ii) positivity for C7-HRP; (iii) disease duration of more than 4 years [6]. The relapse within 1 year after CSA administration is significantly suppressed by the addition of azathioprine (AZA) (72.5% versus 26.7, *P* = .0237, log-rank test). The addition of AZA after CSA treatment significantly reduced the colectomy rate (66.7% versus 30.5%, *P* = .0419, log-rank test). Among CSA responders, AZA naïve patients had significant lower probabilities for colectomies compared to patients with prior AZA treatment (22.5% versus 56.7%, *P* = .0309, log-rank test) [6]. Our result revealed factors affecting the efficacy of CSA therapy for patients with refractory UC. AZA is important agent that maintains disease quiescence once one responds to CSA. However, refractory patients, despite AZA treatment, are more likely to have consequent colectomies. 

In our hospital, low dose of sulfamethoxazole-trimethoprim complex (400 mg of sulfamethoxazole and 80 mg of trimethoprim per day) is administrated prophylactically to the patients who is under treatment with calcineurin inhibitors. Therefore we have no experience of severe infectious diseases such as *Pneumocystis carinii* infection. Among 41 patients, discontinuation of CSA was experienced in one case with renal dysfunction and two cases with liver dysfunction.

### 2.2. Tacrolimus (FK506)

FK506 has similar pharmacological mechanisms to CSA. Fellermann et al. reported the effectiveness of intravenous or oral FK506 for steroid refractory UC patients with doses of 0.01-0.02 mg/kg per day or 0.1-0.2 mg/kg/day, respectively [8]. Ogata et al. have reported the results of a placebo-controlled double blind study which revealed that oral FK506 improved in disease activity for 68.4% in the high trough group compared with 10.0% in the placebo group (*P* < .001) [9]. In the high trough group, 20.0% of patients had clinical remission and 78.9% had mucosal healing. In the open label extension, 55.2% of all patients had an improved disease activity score at week 10. FK506 also had steroid sparing effect. The most common adverse event was mild finger tremor.

In Japan, oral FK506 became an alternative for refractory UC from July in 2009 under national health insurance. Oral FK506 has slower onset of action compared to intravenous CSA. This is because serum level of FK506 reached high trough (10–15 ng/mL) after approximately one week of starting oral FK506. In our experiences, total oral daily dose of FK506 which achieves 10–15 ng/mL is approximately 0.1 mg/kg per day. Therefore, we set our starting dose of FK506 to 0.1 mg/kg per day. Food intake is known to reduce serum level of FK506 resulting from low absorption rate. We recommend oral administration of FK506 one hour prior to each meal. 

In our hospital, intravenous CSA and oral FK506 were used in total 58 moderate to severe refractory cases between 1999 and 2010. Two cases were treated with both CSA and FK506. Excluding these two cases, CSA was used in 47 cases and FK506 was used in 9 cases. Disease activity was measured by Seo's CIDAI. Among 47 CSA cases, 56.5% were severe cases. On the other hand, only 22.2% were severe cases in FK506 cases. In line with this result, the decrease of Seo's CIDAI at one week after the administration of CNI CSA was 55.9 point in CSA cases versus 22.4 point in FK506 cases (*P* < .01). Switching of CNI was tried in two cases due to induction failure (one case from FK506 to CSA, the other case from CSA to FK506). However none of two case was successful. Adverse event of oral FK506 was one case with renal dysfunction. From our experiences, we choose CSA treatment in the critical urgent cases because induction therapy by using oral FK506 usually needs one to achieve efficacious serum levels. 

Anti-TNF*α*-antibody (infliximab) became an alternative for refractory UC from July in 2010 under Japanese national health insurance. As the induction therapy of steroid-refractory ulcerative colitis, there are at least four options in Japan: (1) cytapheresis therapy (including “intensive” regimen), (2) intravenous CSA, (3) oral FK506, and (4) infliximab. However, there are no guidelines of the sequence and timing of these options. We have used infliximab for 5 cases of CNI refractory UC. All of them are showing favorable response and having maintenance therapy. Randomized study comparing intravenous CSA and infliximab is ongoing in the United States. More of such studies are expected to accumulate knowledge on the emerging drugs.

## 3. Azathioprine (AZA) and 6-Mercaptopurine (6-MP)

**Figure 1:**
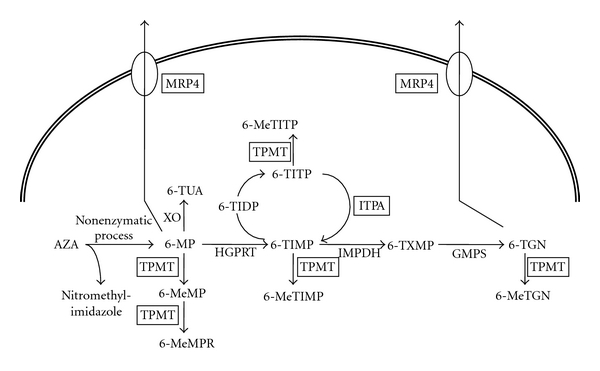
Metabolism and transportation of AZA/6-MP and its metabolites. *XO:* Xanthine oxidase, *TPMT:* thiopurine S-methyltransferase, *HGPRT:* hypoxanthine–guanine phosphoribosyl transferase, *ITPA:* inosine triphosphate pyrophosphatase, *IMPDH:* inosine monophosphate dehydrogenase, *GMPS:* guanosine monophosphate synthetase, *AZA:* azathioprine, *6-MP:* 6-mercaptopurine, *6-TUA: *6-thiouric acid, *6-MeMP:* 6-methylmercaptopurine, *6-MeMPR:* 6-methylmercaptopurine ribonucleotide, *6-TIMP: *6-thioinosine monophosphate, *6-TIDP:* 6-thioinosine diphosphate, *6-TITP:* 6-thioinosine triphosphate, *6-MeTIMP:* 6-methylthioinosine monophosphate, *6-MeTITP:* 6-methylthioinosine triphosphate, *6-TXMP:* 6-thioxanthosine 50-monophosphate, *6-TGN:* 6-thioguanine nucleotide, *6-MeTGN: *6-methylthioguanine nucleotide (redrawn from [7]).

The thioguanine derivative, AZA, is a prodrug of 6-MP that is further metabolized by various enzymes (Figure 1). Although their exact mode of action is still unknown, the mode of action of AZA is thought to be multifactorial: (1) acting as a purine antimetabolite, (2) preventing proliferation of cells involved in the immune response, (3) damage to DNA through the incorporation of thiopurine analogues, resulting in altering lymphocyte function, reduce the number of lamina propria plasma cells, and affect natural killer cell function [10]. 

The thiopurine drugs AZA and 6-MP are the most common drugs used to maintain clinical remission in Crohn's disease and UC [11–13]. These drugs are also important as steroid-sparing agents in chronic active inflammatory bowel diseases. However, concerns remain regarding drug-induced toxicity, such as bone marrow suppression, hepatotoxicity, pancreatitis, fever, rash, and gastrointestinal intolerance [14, 15]. These drugs have to be discontinued due to side effects in about 15–30% of patients [16–19]. In Western countries, an AZA dosage of 2-3 mg/kg is recommended for the treatment of IBD patients [20], but lower doses of AZA (0.6–1.2 mg/kg/day) are used in Japan because of the relatively heightened sensitivity [21]. In our hospital, starting dose of AZA is 25 mg/day/body. Weekly blood test including WBC count, liver function test, and amylase is done until two to three months after AZA introduction. 

The cytotoxic and immunosuppressive properties of AZA/6-MP are mediated by 6-thioguanine nucleotide (6-TGN), a metabolite of AZA/6-MP. 6-TGN incorporates into the DNA, thus leading to DNA breakage and an inhibition of immune cell proliferation [22]. Some patients are more susceptible to bone marrow toxicity while on AZA/6-MP therapy. This susceptibility is genetically dependent on interindividual variations in thiopurine *S*-methyltransferase (TPMT) enzyme activity, based on the genetic polymorphism of low-metabolizing alleles [22]. Several variant *TPMT* alleles of low metabolization have been described recently in many ethnic groups [23]. *TPMT* A719G (*TPMT∗3C*) is associated with intermediate to low TPMT enzyme activity. However, the frequency of *TPMT* A719G is around 2% in the Japanese population. For largely unknown reasons, a subset of other patients who have not inherited the TPMT deficiency also experience thiopurine-induced myelotoxicity. 

Genetic polymorphism of inosine triphosphate pyrophosphatase (ITPase) was also suspected as another factor responsible for thiopurine intolerance. ITPase catalyses the breakdown of inosine triphosphate as part of a futile cycle in the purine metabolic pathway. Genetic ITPase deficiency results in the cellular accumulation of thioinosine triophosphate (TITP) following exposure to thiopurines. Many recent studies have failed to prove an association between the development of thiopurine toxicity and ITPase polymorphism, although a few studies have suggested a role for *ITPase* variants in thiopurine-induced toxicity. 

We have recently elucidated the multidrug-resistance protein 4 (MRP4) polymorphism as a new factor accounting for thiopurine sensitivity in Japanese patients [7]. Of the 279 samples analyzed (44 healthy volunteers and 235 IBD patients), 68 samples showed a heterozygote of *MRP4* G2269A and 7 carried a homozygote. The allelic frequency of MRP4 G2269A was 14.7%. In 130 IBD patients treated with AZA/6-MP, the white blood cell count was significantly lower in the patients with the *MRP4* variant alone (*n* = 26) than in patients with a wild allelotype (*n* = 74) (*P* = .014) or in the patients with the *ITPase* variant alone (*n* = 22) (*P* = .0095). The 6-TGN levels were significantly higher in patients with the *MRP4* variant alone than in patients with the wild allelotype (*P* = .049). Of the 15 patients who experience leucopenia (<3 × 109/L), 7 patients carried the *MRP4* variant. The odds ratio of carrying the *MRP4* variant alone and having leucopenia was 3.30 (95% confidence interval 1.03–19.57, *P* = .036).

## 4. Conclusion

We have described the characteristics and usage of immunomodulators and immunosuppressants focusing on Japanese patients. As for immunosuppressants, effectiveness of intravenous CSA is similar to the Western countries in remission induction therapy. On the other hand, oral FK506 which needs at least one week to exert effectiveness is also an alternative therapy in remission induction. Immunomodulators such as AZA and 6-MP need special attention when determining a dose. The metabolism of AZA and 6-MP is largely affected by various enzymes. Japanese patients need less AZA/6-MP to obtain enough 6-TGN concentration compared to Westerners. As medical options increase, decisions about the sequence and timing of therapy become more difficult. Consequently a therapeutic strategy is necessary, taking the measure of not only the disease severity and course but also the selected therapeutic options. Further evidence should be accumulated to obtain the rapid induction of clinical remission, steroid-free maintenance of clinical remission, avoidance of complications, hospitalizations and surgeries, and improvement of quality of life.

## References

[1] Sandborn WJ (1995). A critical review of cyclosporin therapy in inflammatory bowel disease. *Inflammatory Bowel Disease*.

[2] Actis GC, Ottobrelli A, Pera A (1993). Continuously infused cyclosporine at low dose is sufficient to avoid emergency colectomy in acute attacks of ulcerative colitis without the need for high-dose steroids. *Journal of Clinical Gastroenterology*.

[3] Lichtiger S, Present DH, Kornbluth A (1994). Cyclosporine in severe ulcerative colitis refractory to steroid therapy. *The New England Journal of Medicine*.

[4] Cohen RD, Brodsky AL, Hanauer SB (1999). A comparison of the quality of life in patients with severe ulcerative colitis after total colectomy versus medical treatment with intravenous cyclosporin. *Inflammatory Bowel Diseases*.

[5] Seo M, Okada M, Yao T, Ueki M, Arima S, Okumura M (1992). An index of disease activity in patients with ulcerative colitis. *The American Journal of Gastroenterology*.

[6] Bamba S, Tsujikawa T, Inatomi O (2010). Factors affecting the efficacy of cyclosporin A therapy for refractory ulcerative colitis. *Journal of Gastroenterology and Hepatology*.

[7] Ban H, Andoh A, Imaeda H (2010). The multidrug-resistance protein 4 polymorphism is a new factor accounting for thiopurine sensitivity in Japanese patients with inflammatory bowel disease. *Journal of Gastroenterology*.

[8] Fellermann K, Tanko Z, Herrlinger KR (2002). Response of refractory colitis to intravenous or oral tacrolimus (FK506). *Inflammatory Bowel Diseases*.

[9] Ogata H, Matsui T, Nakamura M (2006). A randomised dose finding study of oral tacrolimus (FK506) therapy in refractory ulcerative colitis. *Gut*.

[10] Nielsen OH, Vainer B, Rask-Madsen J (2001). Review article: the treatment of inflammatory bowel disease with 6-mercaptopurine or azathioprine. *Alimentary Pharmacology and Therapeutics*.

[11] Adler DJ, Korelitz BI (1990). The therapeutic efficacy of 6-mercaptopurine in refractory ulcerative colitis. *The American Journal of Gastroenterology*.

[12] Sandborn W, Sutherland L, Pearson D, May G, Modigliani R, Prantera C (2000). Azathioprine or 6-mercaptopurine for inducing remission of Crohn’s disease. *Cochrane Database of Systematic Reviews*.

[13] Hibi T, Ogata H (2006). Novel pathophysiological concepts of inflammatory bowel disease. *Journal of Gastroenterology*.

[14] Gearry RB, Barclay ML, Burt MJ, Collett JA, Chapman BA (2004). Thiopurine drug adverse effects in a population of New Zealand patients with inflammatory bowel disease. *Pharmacoepidemiology and Drug Safety*.

[15] Van Dieren JM, Hansen BE, Kuipers EJ, Nieuwenhuis EE, Van Der Woude CJ (2007). Meta-analysis: inosine triphosphate pyrophosphatase polymorphisms and thiopurine toxicity in the treatment of inflammatory bowel disease. *Alimentary Pharmacology and Therapeutics*.

[16] Gisbert JP, Gomollón F (2008). Thiopurine-induced myelotoxicity in patients with inflammatory bowel disease: a review. *The American Journal of Gastroenterology*.

[17] Takatsu N, Matsui T, Murakami Y (2009). Adverse reactions to azathioprine cannot be predicted by thiopurine S-methyltransferase genotype in Japanese patients with inflammatory bowel disease. *Journal of Gastroenterology and Hepatology*.

[18] Lees CW, Maan AK, Hansoti B, Satsangi J, Arnott IDR (2008). Tolerability and safety of mercaptopurine in azathioprine-intolerant patients with inflammatory bowel disease. *Alimentary Pharmacology and Therapeutics*.

[19] Winter JW, Gaffney D, Shapiro D (2007). Assessment of thiopurine methyltransferase enzyme activity is superior to genotype in predicting myelosuppression following azathioprine therapy in patients with inflammatory bowel disease. *Alimentary Pharmacology and Therapeutics*.

[20] Lichtenstein GR, Sbreu MT, Cohen R, Tremaine W (2006). American gastroenterological association Institute technical review on corticosteroids, immunomodulators, and infliximab in inflammatory bowel disease. *Revista de Gastroenterología de México*.

[21] Hibi T, Naganuma M, Kitahora T, Kinjyo F, Shimoyama T (2003). Low-dose azathioprine is effective and safe for maintenance of remission in patients with ulcerative colitis. *Journal of Gastroenterology*.

[22] Derijks LJ, Gilissen LP, Engels LG (2006). Pharmacokinetics of 6-thioguanine in patients with inflammatory bowel disease. *Therapeutic Drug Monitoring*.

[23] Hiratsuka M, Inoue T, Omori F, Agatsuma Y, Mizugaki M (2000). Genetic analysis of thiopurine methyltransferase polymorphism in a Japanese population. *Mutation Research—Fundamental and Molecular Mechanisms of Mutagenesis*.

